# Under Pressure That Splits a Family in Two. The Case of Lipocalin Family

**DOI:** 10.1371/journal.pone.0050489

**Published:** 2012-11-27

**Authors:** Stephane Marchal, Anna Marabotti, Maria Staiano, Antonio Varriale, Thomas Domaschke, Reinhard Lange, Sabato D’Auria

**Affiliations:** 1 INSERM U710, University of Montpellier 2, Montpellier, France; 2 Institute of Biomedical Technologies, CNR, Segrate, Milano, Italy; 3 Laboratory for Molecular Sensing, IBP-CNR, Naples, Italy; Russian Academy of Sciences, Institute for Biological Instrumentation, Russian Federation

## Abstract

The lipocalin family is typically composed of small proteins characterized by a range of different molecular recognition properties. Odorant binding proteins (OBPs) are a class of proteins of this family devoted to the transport of small hydrophobic molecules in the nasal mucosa of vertebrates. Among OBPs, bovine OBP (bOBP) is of great interest for its peculiar structural organization, characterized by a domain swapping of its two monomeric subunits. The effect of pressure on unfolding and refolding of native dimeric bOBP and of an engineered monomeric form has been investigated by theoretical and experimental studies under pressure. A coherent model explains the pressure-induced protein structural changes: i) the substrate-bound protein stays in its native configuration up to 330 MPa, where it loses its substrate; ii) the substrate-free protein dissociates into monomers at 200 MPa; and iii) the monomeric substrate-free form unfolds at 120 MPa. Molecular dynamics simulations showed that the pressure-induced tertiary structural changes that accompany the quaternary structural changes are mainly localized at the interface between the monomers. Interestingly, pressure-induced unfolding is reversible, but dimerization and substrate binding can no longer occur. The volume of the unfolding kinetic transition state of the monomer has been found to be similar to that of the folded state. This suggests that its refolding requires relatively large structural and/or hydrational changes, explaining thus the relatively low stability of the monomeric form of this class of proteins.

## Introduction

Lipocalins represent a big family of proteins exhibiting a large functional diversity. In fact, although they have been formerly classified as transport proteins, the members of this family fulfill a variety of different functions, including transport of small ligands, cryptic coloration, olfaction, and the enzyme synthesis of prostaglandins; in addition, the lipocalins have also been implicated in the regulation of the immune response and the mediation of cell homoeostasis [1]. Members of the lipocalins family are typically small proteins characterized by a range of different molecular recognition properties such as their ability to bind small and mainly hydrophobic molecules, their binding to specific cell-surface receptors and their involvement in the formation of macromolecular complexes [2]. A low level of similarity of protein sequences is present in the lipocalin family, but three short conserved sequence motifs are recognizable and constitute an hallmark to discriminate among the so-called “kernel” lipocalins (those with all three sequence motifs) and the “outliers” (those with only one or two motifs) [3]. On the contrary, a conserved structural pattern is common both for kernel and outlier lipocalins: a highly symmetrical β-barrel structure formed by an antiparallel β-sheet, calyx-shaped, with β-strands connected by short β-hairpins (except the first loop L1, between strands A and B, which is larger and flexible and functions as a lid for the open-end of the barrel). In addition, an α-helix is located between strand H and the short terminal strand I. The β-barrel is stabilized by one, two or three disulfide bridges and by transversal hydrogen bonds that connect the strands [2], [4].

**Figure 1:**
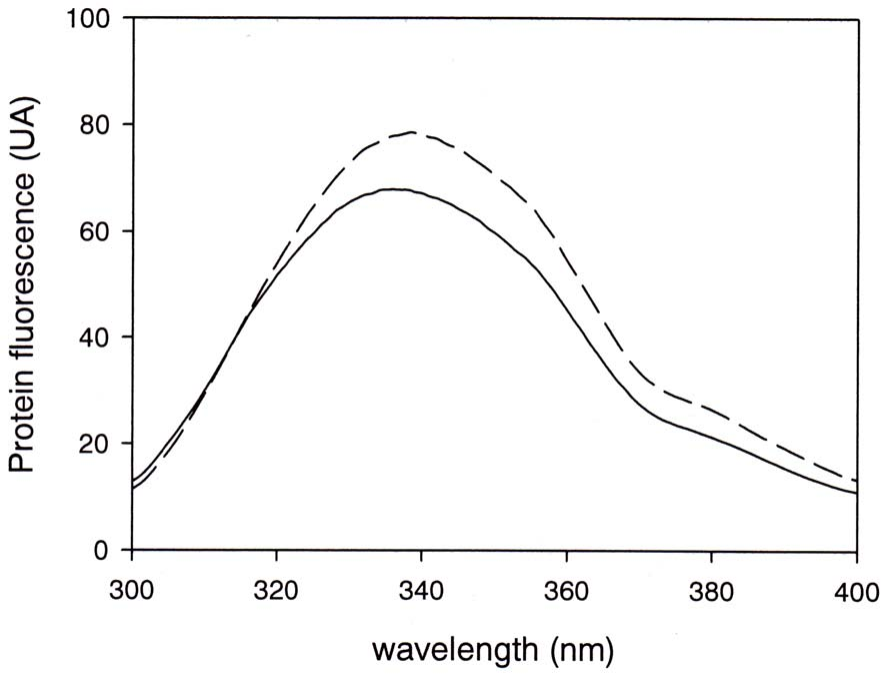
Intrinsic Fluorescence of OBP. Intrinsic fluorescence emission spectra of native dimer OBP in the absence (dashed trace) and in the presence of 1.5 M GdnHCl (solid line) at 0.1 MPa.

Odorant binding proteins (OBPs) are small outlier lipocalins expressed in nasal mucosa that show a broad specificity towards hydrophobic molecules of medium size, such as odorants [5]. To date, several OBPs are known, and for some of them, the three-dimensional structure is available. The first structure solved by X-ray crystallography was that of bovine OBP (bOBP) [6], [7], that, contrarily to other monomeric structures of OBP from pig [8], rat [9] and insect [10], is a dimer characterized by a “domain swapping”, in which the helix of the one monomer is located where the helix of the other monomer would be in a classic monomeric lipocalin. This particular fold is linked to the fact that bOBP lacks two conserved cysteine residues that form a disulfide bridge in the rest of the family, and also lacks a conserved glycine residue in position 121. In fact, the structure of the triple mutant in which the glycine residue and the disulfide bridge were reinserted, shows a monomeric fold very similar to that of porcine OBP [11]. bOBP, however, is not the only lipocalin protein showing domain swapping: another available structure of a domain-swapped lipocalin is that of queen bee pheromone-binding protein, in which the presence of the domain swapping is controlled by pH and it is disrupted by a single mutation in position 35 [12]. In addition, the tendency to aggregation seems to be a typical feature of lipocalin family [4]. Indeed, the all-β structure allows the tight packing of lipocalins, and factors such as low pH values or high calcium concentrations favor this phenomenon [13]. Also the presence of the ligand can influence the protein oligomerization state, and *vice versa* the level of aggregation can influence ligand binding [14].

Recently, considerable efforts are being undertaken for elucidating the mechanisms of protein folding, misfolding, aggregation and dissociation. The reason of the general interest for these reactions is their medical and industrial importance. Indeed, the major age-related neurodegenerative diseases, such as Alzheimer, Parkinson and Creutzfeldt-Jakob disease, are related to protein misfolding and aggregation [15], [16], [17], [18], and recently, domain swapping has been focused as a main mechanism for amyloid-fibril formation [19]. Furthermore, the knowledge of mechanisms for protein aggregation is of outstanding importance for biotechnological applications that are depending on the structural and functional integrity of proteins, and to avoid problems such as immunogenicity of protein bio/pharmaceuticals related to aggregation phenomena [20] (and references therein). The mechanisms of these reactions are generally studied by analyzing the effects of perturbing the native structures of model proteins *via* increasing temperature or adding chemical denaturants, such as urea or guanidinium hydrochloride (GdnHCl).

**Figure 2:**
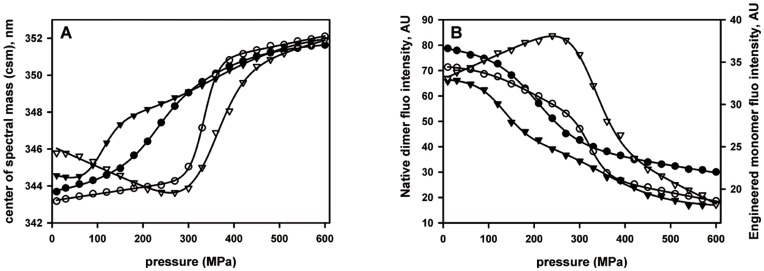
Effect of the binding of the natural substrate 1-octen-3-ol on the OBP stability under pressure. Spectral transition curves were obtained by plotting the fluorescence center of spectral mass (panel A) and the maximum intensity of protein fluorescence emission (panel B) as a function of pressure. OBP proteins (dimer: circle, monomer: triangle) in 1.5 M GdnHCl, 20 mM Tris buffer pH 7.4 were incubated in the absence (•,▾) or in the presence (○,▿) of 1-octen-3-ol for 1 hour at 25°C before application of pressure. The best fit was made according to a two-step transition and the corresponding values are reported in Table 1.

Since some decades, an elegant alternative method has been developed and gained importance, using pressure as perturbing tool [21], [22], [23]. Pressure is a particularly useful parameter, because its use provides unique information concerning protein packing and hydration properties, which both are important in protein tertiary and quaternary structural changes [24]. Indeed, high pressure induces structural changes that reduce the overall volume of a reaction system [25]. Protein unfolding and aggregation reactions are usually accompanied by a decrease in volume. This is believed to be caused by the combined effects of electrostriction of water molecules around newly exposed charged and polar groups, the decrease in partial volume of hydrophobic residues upon transfer from a nonpolar protein interior to water, and the elimination of packing defects [26]. Moreover, high pressure processes are increasingly used to remove and refold protein aggregates or to facilitate enzymatic reactions at industrial scales [27].

In the case of dissociation and unfolding of oligomeric proteins, an intriguing question is to determine the mechanism and the sequence of these processes. In this work, we have used high pressure, in association with the use of chemical denaturants, to elucidate this question. The targets of these studies were the native dimeric form of bOBP and the triple “deswapped” mutant of this protein in monomeric form, in the absence or presence of the ligand. Because of their structural peculiarities, these two proteins can be considered representatives of the whole lipocalin class, that in its turn can be considered a model class of proteins prone to aggregation. The application of this multidisciplinary approach led to the elucidation of the sequence of pressure-induced protein structural changes and unraveled some reasons of the noticeable structural stability of this class of proteins.

## Materials and Methods

### Reagents

All chemicals were of the highest purity grade available from commercial sources and used without further purification. N-acetyl-tryptophanamide (NATA) and Tris were obtained from Sigma Chemical Co (St. Louis, MO). Glycerol was obtained from Merck. 1-Aminoanthracene (AMA) and 1-octen-3-ol were from Sigma. Water, doubly distilled over quartz, was purified by using a Milli-Q Plus system (Millipore corp., Bedford, MA). All glassware used for sample preparation was conditioned in advance by standing for 24 hr in 10% HCl suprapur (Merck, Darmstadt). Purification and functionality of wild-type and mutant bovine OBPs were tested with 1- amino-anthracene (AMA).

**Figure 3:**
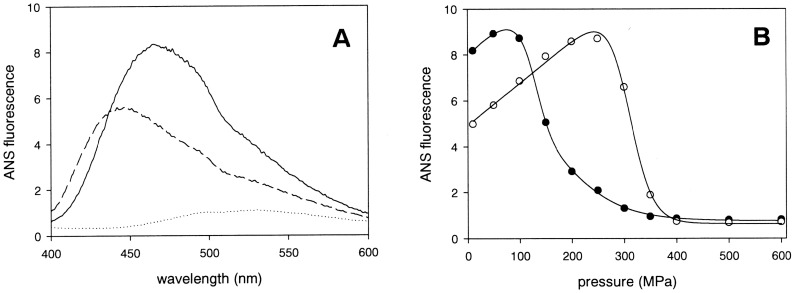
Protein fluorescence characterization in the presence of ANS. Panel A: ANS fluorescence emission spectra of dimer (full line) and monomer (dashed line) bOBP in the absence of substrate at 0.1 MPa. In the absence of proteins, the ANS probe exhibits only low fluorescence (dotted line). Panel B: ANS fluorescence intensity at 450 nm of the dimer OBP protein as a function of pressure in the absence (filled symbols) or in the presence (empty symbols) of 1 mM 1-octen-3-ol, respectively.

#### Wild-type and mutant bovine OBPs

A 6×His affinity tag was placed at the N-terminal of the wild-type and mutant bOBP form by PCR using specific primers. The fused cDNAs were sub-cloned in the expression vector pT7-7 and the expression of the protein, in BL21-DE 3 *E. coli,* was realized as reported above for the recombinant forms of bOBP. The purification of the protein was obtained by affinity chromatography with a Ni-NTA Agarose (Quiagen, Germany) according to the manufacturer’s instructions, followed by a second chromatographic step on the anion exchange column Resource Q (Amersham Biosciences, Italy), in FPLC [28], [29]. Functionality of the two different OBP forms was determined by direct titrations using the fluorescent ligand AMA [29]. Briefly, 1.0 ml samples of 1.0 µM wild-type and mutant bOBP, in 20 mM Tris-HCl buffer pH 7.8, were incubated overnight at 4°C in the presence of increasing concentrations of AMA (0.156–10 µM). Fluorescence emission spectra between 450 and 550 nm were recorded with a Perkin-Elmer LS 50 luminescence spectrometer (excitation and emission slits of 5 nm) at a fixed excitation wavelength of 380 nm and the formation of the AMA-OBP complex was followed as an increase of the fluorescence emission intensity at 480 nm. The dissociation constants of the AMA-OBP complexes were determined from the hyperbolic titration curves using the nonlinear fitting program of Sigma Plot 5.0 (Cambridge Soft. Corp., Cambridge, MA, USA). The concentrations of the AMA-OBP complexes were determined on the basis of emission spectra obtained incubating AMA (0.1–10 µM) with saturating amounts of both bOBP forms [29].

### MD Simulations

Molecular dynamics (MD) simulations on bOBP in the presence of its ligand 1-octen-3-ol and in different pressure conditions were performed with Gromacs v. 4.5.4. [30]. The GROMOS96 43a1 force field [31] was used throughout simulations. The starting structure was retrieved from PDB database (code: 1G85) [32]. The topology of the ligand was created with the aid of the server PRODRG 2.5Beta [33], with careful manual revision of charges. The dimeric protein was simulated in a rhombic dodecahedral box, fixing a distance of 1.5 nm between the protein (centered in the box) and the box walls, so that the dimensions of the box are set to the diameter of the protein plus twice the specified distance. Approximately 30000 water molecules (SPC model) [34] were added to each system, which was neutralized with 19 Na^+^ ions. Simulations were performed under periodic boundary conditions.

**Table 1:** Thermodynamic parameters calculated from pressure unfolding curves of OBP at 25°C in the presence of 1.5 M GdnHCl.

Recorded by increasing pressure			
*Dimer*	ΔV, ml.mol^−1^	ΔGu, kJ.mol^−1^	P_1/2_, MPa
Csm	−34.18+/−1.24	8.37+/−0.34	245
fluorescence intensity	−58.54+/−3.59	11.98+/−0.97	204
ANS fluorescence	−45.92+/−4.52	7.11+/−1.36	154
***Dimer+ligand***			
Csm	−172.60+/−5.80	57.56+/−1.93	333
Fluorescence intensity	−138.70+/−8.95	44.69+/−2.90	322
ANS fluorescence	−114.10+/−9.10	35.19+/−4.00	308
***Monomer***			
Csm	−124.6+/−5.53	13.20+/−0.83	105
Fluorescence intensity	−88.98+/−8.27	11.96+/−1.20	134
ANS fluorescence	−92.17+/−4.44	10.73+/−0.57	116
***Monomer+ligand***			
Csm	−82.68+/−6.22	29.15+/−2.12	352
Fluorescence intensity	−88.73+/−2.09	28.65+/−2.09	323
**Recorded by decreasing pressure**			
***Dimer***	**ΔV, ml.mol^−1^**	**ΔGu, kJ.mol^−1^**	**P_1/2_, MPa**
Csm	−61.47+/−2.95	4.52+/−0.02	73.53
fluorescence intensity	−78.20+/−3.75	4.76+/−0.2	60.86
***Dimer+ligand***			
Csm	− 95.71+/−9.99	12.63+/−1.21	131.96
fluorescence intensity	−72.58+/−9.97	8.52+/−1.27	117.38
***Monomer***			
Csm	−141.90+/−15.61	11.96+/−1.36	84.28
***Monomer+ligand***			
Csm	−103.30+/−10.68	12.04+/−1.32	116
fluorescence intensity	−96.97+/−15.41	9.89+/−1.93	102

The complex was first energy minimized using the Steepest Descent method, with a gradient limit of 500 kJ/mol/nm, using P-LINCS [35] to constrain all bonds. Then, the system was submitted to a 20 ps-long MD simulation with position restraints using the NVT ensemble, at 25°C with a velocity rescaling thermostat with a stochastic term [36]. After then, three 100 ps-long different position-restrained simulations with NPT ensemble were set up at three different pressures (0.1, 250, 600 MPa), using Berendsen’s method [37]. In all cases, a time step of 2 fs was used, long-range electrostatics were handled using the PME method [38], and cut-offs were set at 1.0 nm for Coulombic short-range interactions and at 1.4 nm for van der Waals short-range interactions. Finally, the full MD simulations were carried out with the same settings adopted for the short NPT simulation, but without any position restraints. For each system simulated at different pressure, a 100 ns-long simulation was obtained. At the end of simulations, systems stability was verified analyzing the energy components and the convergence of the root mean square deviation (RMSD) of the structures in the trajectory compared to the starting structure. Moreover, to verify the absence of non-physical self-interactions between periodic images of the protein during simulations, minimum distance between periodic images was evaluated.

Analyses of MD simulations were conducted using the software from GROMACS package. The cluster analysis was made using the clustering method of Daura [39], with a cut-off of 0.25 nm. The central structure of each cluster was selected as representative of the cluster itself and saved in a.pdb file, not including hydrogen atoms. The variation of secondary structure was analyzed using DSSP [40] both on the whole trajectory and on these representative.pdb structures. Results were visualized and elaborated with the aid of the freeware program Grace (http://plasma-gate.weizmann.ac.il/Grace).

**Figure 4:**
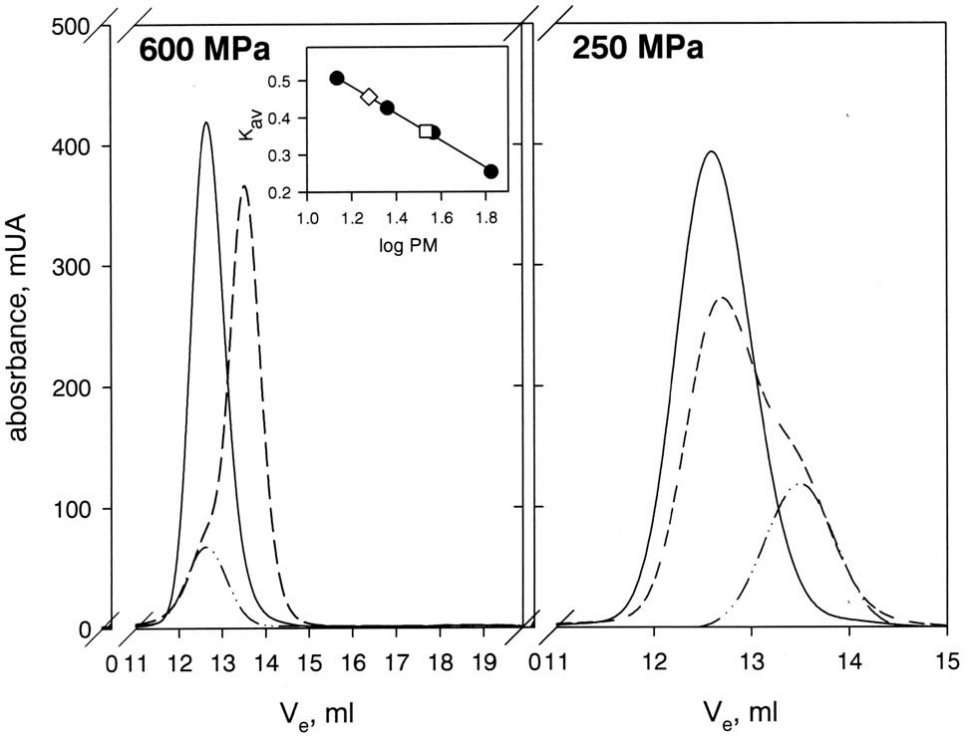
Structural stability of dimer OBP after pressure treatment analyzed by size exclusion chromatography. Chromatograms of the dimer protein OBP in the absence of substrate before (solid line) and after (dashed line) pressure treatment up to 600 MPa and 250 MPa. The dash-dot-dot line represents the relative contribution of the monomer state of OBP assuming a Gaussian profile of elution for each oligomeric state of the protein. (*Inset*) The quantification of the monomer species was evaluated using bovine serum albumin (67 kDa), ovalbumin (36.9 kDa), chymotrypsinogen (23 kDa) and ribonuclease A (13.7 kDa) as standard proteins (filled circles). From the calculated partition coefficient K_AV_, molecular masses of 34.8 kDa and 18.97 kDa were determined for the initial dimer form (open square) and the pressure-induced dissociated form (open diamond), respectively.

### Fluorescence Measurements Under High Pressure

The bovine dimer and the engineered monomer proteins were diluted to a concentration of 0.36 mg/mL in 1.5 M GdnHCl 10 mM Tris-HCl buffer at pH 7.4 in the absence or in the presence of 1 mM 1-octen-3-ol. The fluorescence experiments were carried out at 25°C using an SLM Series 2 luminescence spectrometer (Aminco Bowman) modified to accommodate a high pressure cell [41]. For equilibrium studies tryptophan fluorescence was excited at 280 nm, using a bandwidth of 4 nm. Emission (accumulation of three scans) was collected between 300 and 400 nm with a bandwidth of 4 nm. 8-Anilino-1-naphthalenesulfonate (ANS) fluorescence was measured by exciting at 350 nm (slit of 4 nm) and recording emission spectra (4-nm slit) from 400 to 600 nm. The ANS concentration was 700 µM. For kinetic studies, tryptophan fluorescence intensity was recorded at 340 nm (4-nm slit) and excited at 280 nm using a 4-nm slit.

**Figure 5:**
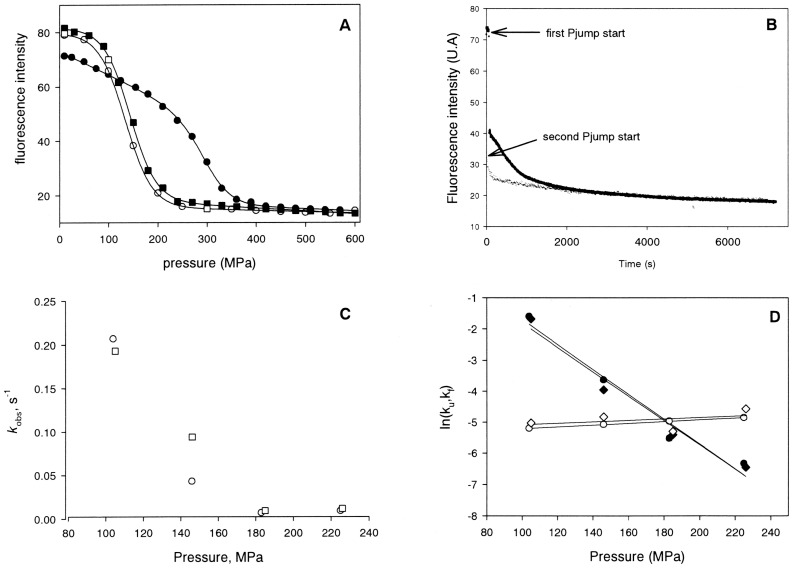
Successive pressurization cycles of OBP binding protein in the presence of substrate. A) The protein fluorescence intensity at 338 nm is shown for increasing (filled symbols) and decreasing (empty symbols) pressures in a first (circles) and a second (squares) pressurization cycle. B) Pressure-jump induced relaxation kinetics. The p-jumps were from 185 to 350 MPa. The upper and lower traces reflect the kinetics in the first (close circles) and second pressurization cycle (open circle), respectively. C) Pressure-dependence of *k*_obs_ in the first and second pressurization cycles. The rate constants were determined by fitting the kinetic traces of 40 MPa downward pressure jumps to mono-exponential decays for the first (open circle) and the second (open square) cycles. D) Pressure dependence of the individual rate constants k_f_ (filled symbols) and k_u_ (empty symbols) of relaxation kinetics induced by downward pressure-jumps in the first (circles) and second (diamonds) pressurization cycles.

### Pressure-induced Equilibrium Unfolding Transitions

Following each pressure increment/decrement (steps of 30 MPa), the time of equilibration was fixed to 5 min before spectral recording. The pressure-induced fluorescence spectral changes were quantified by determining the emission intensity, *I*, at a characteristic maximum wavelength and the center of spectral mass, csm, using equation 1:
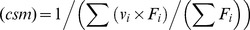
(1)where *F_i_* is the intensity of fluorescence emitted at a wavenumber ν*_i_*. The *csm* parameter reflects the mean exposure of tryptophan residues to water [42].

The thermodynamic parameters were evaluated by fitting the intensity pressure profiles to equation 2:
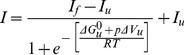
(2)where *I*_f_ and *I*_u_ are the fluorescence intensities of the folded and unfolded states, respectively, and *I* the observed fluorescence intensity at pressure *p*; ΔG^0^_u_ and ΔV_u_ are the free energy and volume changes of unfolding at 0.1 MPa, respectively [25]. Alternatively, the thermodynamic parameters were evaluated from the csm pressure profiles in an analogous way, replacing *I* by *csm* in equation 2.

**Figure 6:**
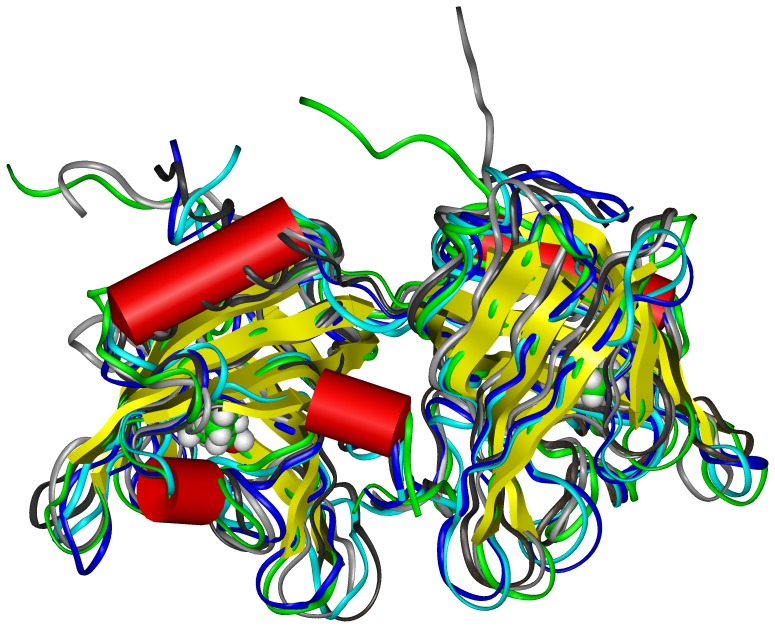
Super-impositions of the representative structures for each cluster in the different simulations. At 0.1 MPa (cluster 1: dark grey, cluster 2 : light grey), 250 MPa (cluster 1: blue, cluster 2: cyan) and 600 MPa (orange) to the crystallographic structure of bOBP (green). Backbone is represented as a ribbon, secondary structures are represented as cylinders (helices) or flat ribbon (β-strands). The ligand 1-octen-3-ol is shown in CPK mode and color coded: carbon green, oxygen red, hydrogen white.

### Pressure-jump-induced Kinetics

Pressure-jumps were performed by using a homemade p-jump device connected to the high pressure optical cell placed in the abovementioned fluorescence spectrophotometer [41]. Pressure-jumps (dead-time <5 ms) were carried out by opening an electrically driven pneumatic valve localized between the high pressure optical cell and a ballast tank.

### Determination of Kinetic Parameters from Relaxation Profiles

After each p-jump the relaxation profiles of the unfolding/folding reaction were fitted to a single exponential or to double (sequential) decays, according to equations 3 and 4:
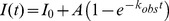
(3)

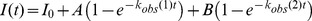
(4)where *I*(t) and *I*_0_ are the fluorescence intensities at time t and at time 0, A and B are the phase amplitudes, and *k*_obs_ is the measured rate constant at the final pressure *p*. The individual rate constants of the folding/unfolding reaction:
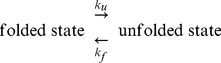
(5)were determined from single exponential kinetics and from the fast phase in cases of two-exponential decays, according to equations 6 and 7:



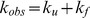
(6)

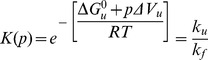
(7)


**Table 2:** DSSP analysis made on the representative structures for each cluster of the three MD simulations.

Secondary structure code	Cluster 10.1 MPa	Cluster 20.1 MPa	Cluster 1250 MPa	Cluster 2250 MPa	Cluster600 MPa
Helices^a^	14,70	15,34	13,42	14,38	15,02
β-strands^b^	44,73	44,73	46,65	42,49	42,17
Others^c^	22.36	23,33	23,33	23.65	23,01
Random^d^	18,21	16,61	16,62	19,49	19,81

a DSSP codes H+G+I.

b DSSP code E.

c DSSP codes B+S+T.

d structures not identified by DSSP code.

where *K*(*p*) is the equilibrium constant at pressure p, ΔG^0^_u_, and ΔV_u_ are the free energy and volume changes of unfolding obtained from equilibrium experiments and p is the final pressure of each jump. Linear plots of ln*k*_f_ and ln*k*_u_ versus the final pressure of each jump allowed to determine ΔV^≠^_f_ and ΔV^≠^_u_, the activation volumes for folding and unfolding, respectively, according to equations 8 and 9:



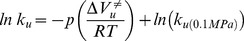
(8)

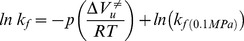
(9)


### Native Molecular Mass Determination

The molecular mass of the bovine dimer protein before and after pressure treatment (250 or 600 MPa) was performed by gel filtration using a Superose 12 FPLC column (Pharmacia) at 4°C equilibrated with 1.5 M GdnHCl, 10 mM Tris-HCl pH 7.4 buffer containing 150 mM KCl at a rate flow of 0.5 ml/min. Samples were centrifuged at 16,000 g for 40 min and 100 µl were loaded onto the column. For calibration, the following molecular mass standards (Sigma), expressed in Da were used: β-amylase (200,000), alcohol dehydrogenase (150,000), albumin (66,000), carbonic anhydrase (29,000), cytochrome C (12,400) and aprotinin (6,500). The void and total volumes of 8 and 20.8 ml, respectively, were determined with cytidine and dextran blue dye for the calculation of the distribution coefficient *K*_av_.

**Figure 7:**
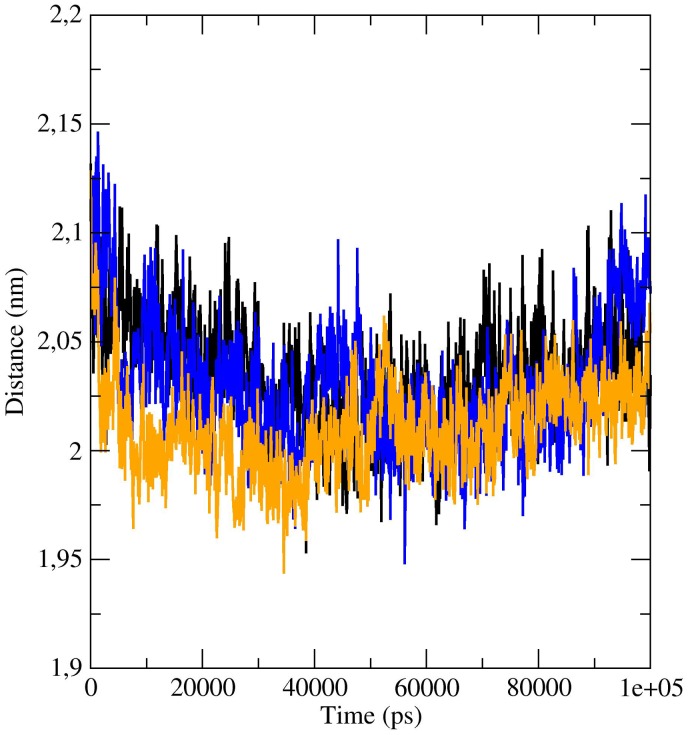
Analysis of the distances between the center of mass of the two subunits during the simulation time. Variation of the distances of the center of mass of the two subunits of bOBP during the time, for simulations at 0.1 MPa (black), 250 MPa (blue) and 600 MPa (orange).

## Results and Discussion

The main goal of this work was to study the mechanism of dissociation and unfolding of bOBP, representing the lipocalin family, with an emphasis on the role of substrate binding. To pursue this goal, we used the following methodological approach: a) high pressure fluorescence spectroscopy measurements. Indeed, for many oligomeric proteins, high pressure can be used to force their dissociation and/or unfolding; b) compare the results obtained on the native dimeric bOBP with those obtained with a genetically modified monomeric bOBP; c) molecular dynamics simulation experiments on native bOBP at high pressure.

### Fluorescence Spectroscopy Experiments at High Pressure

At 25°C, under our experimental conditions the native protein showed a fluorescence emission spectrum centered around 340 nm, reflecting a rather non-polar microenvironment of its six tryptophan residues. Applying pressure up to 600 MPa left the protein structure intact. Furthermore, no significant fluorescence spectral changes could be detected. Therefore we decided to destabilize the protein structure by the addition of 1.5 M GdnHCl, a concentration that had been reported to be sub-denaturing for this protein [43]. Indeed, as shown in Figure 1, in the presence of GdnHCl only minor fluorescence spectral changes were observed at atmospheric pressure. But, as reported below, major changes were observed at higher pressure. From here on, all experiments were therefore conducted in the presence of 1.5 M GdnHCl.

### Intrinsic Fluorescence Spectral Changes at High Pressure

As shown in Figure 2A, the center of spectral mass (csm) of native bOBP in the absence of ligand underwent a pressure dependent 2-state transition from about 344 to 351 nm, with a transition pressure of p_1/2_ = 245 MPa. In the presence of 1 mM substrate (1-octen-3-ol), this transition was strongly shifted to higher pressure, with a p_1/2_ of 333 MPa. Analysis of spectral amplitude at 338 nm, the wavelength of maximum fluorescence intensity of the native protein at atmospheric pressure, showed a pressure-dependent fluorescence quenching that was equivalent to the evolution of the center of spectral mass (Figure 2B). Up to about 250 MPa, these spectral changes were totally reversible. However, they became irreversible when the protein was subjected to higher pressure. Therefore, any thermodynamic interpretation can be considered only as indicative, depending on the spectral recording procedure. In order to be able to compare the pressure dependent spectral behavior of the proteins, all spectra were therefore recorded strictly in the same way, allowing 5 minutes of equilibration before each data collection (see also Materials and Methods section). To decipher the sequential mechanism of the pressure-induced unfolding of the dimer OBP protein, an engineered monomer variant of OBP was also used as a protein model (see Materials and Methods). Again, as for the dimer, single major two-state spectral transitions were observed (Figures 2A, 2B). In addition, a minor additional spectral transition seems to be present at pressures around 350 MPa. As in the case of the dimeric protein, substrate binding increased considerably the stability of the protein. As we can see in Figure 2, this stabilizing effect of substrate was larger in the case of monomeric than for dimeric protein. Hence, the stability of the dimer was larger than that of the monomer in the absence of substrate, and lower than that of the monomer in the presence of substrate.

### ANS Binding Under High Pressure

In addition to intrinsic protein (tryptophan) fluorescence, we also used the environment-sensitive fluorescent probe ANS to investigate the pressure-induced protein conformational changes. ANS is known to bind to relatively large and structured unpolar protein surface domains [44]. As shown in Figure 3A, in the absence of substrate, the dimeric and the monomeric proteins bound the ANS probe at 0.1 MPa. This indicates that the monomeric as well as the dimeric proteins possess large hydrophobic surface domains. As shown in Figure 3B, in the presence of substrate, the dimeric protein still bound ANS, although to a lesser degree. However, the substrate bound monomeric protein did not bind to ANS, indicating that its hydrophobic surface domain was no more accessible to ANS (data not shown). The reduced ANS binding of substrate bound dimeric bOBP is therefore suggestive of the binding of only one substrate molecule binding to the dimer. In the presence of 1-octen-3-ol, the relatively low ANS fluorescence intensity of the dimeric protein increases as a function of pressure up to 250 MPa to attain the same level as that of dimeric protein in the absence of substrate. This may readily be explained by a pressure-induced dissociation of substrate. At higher pressures, the fluorescence becomes strongly quenched, similarly to the situation observed in the absence of substrate.

The pressure-induced ANS fluorescence quenching is explained by dissociation of ANS from the protein. The thermodynamic parameters deduced from these processes are listed in Table 1, where they are compared to the parameters deduced from pressure-induced intrinsic protein fluorescence changes. Clearly, the two independent spectroscopic analyses, tryptophan and ANS fluorescence, yielded very similar results. Although the absolute values of the parameters must be regarded with caution, because of the partial irreversibility of the spectral changes, they may nevertheless be useful for comparative reasons. From Table 1 it appears that the spectral changes observed in pressure-increasing experiments are falling into three classes, distinguishable by their p_1/2_ values: i) mutant monomeric protein, in the absence of substrate with p_1/2_ of about 120 MPa; ii) native dimeric protein, in the absence of substrate with p_1/2_ of about 200 MPa; iii) both proteins (mutant monomer and native dimer), in the presence of substrate with p_1/2_ of about 330 MPa. In contrast, the spectral changes observed in pressure-decreasing experiments are all showing more or less the same value of p_1/2_ of about 100 MPa. Finally, whether mutant monomer or native dimer, the substrate dissociates at 330 MPa. These results indicate that the protein unfolding occurs at 120 MPa unless it is in its substrate bound and/or dimeric protected form. An additional result that arises from the above spectroscopic experiments is the indication that at 200 MPa the protein dimer dissociates into monomers unless it is in the presence of the substrate. In order to confirm this interpretation, the effect of exposure to pressures of 250 and 600 MPa to the protein oligomeric state was evaluated by size exclusion chromatography.

### Effect of Pressure on the Protein Oligomeric State

As previously described [45], the molecular mass of OBP calculated from the elution profile for the dimer under sub-denaturing conditions is compatible to that of the native dimeric protein (34.8 kDa versus 37.0 kDa). As shown in Figure 4, after pressure treatment at 250 MPa, in the absence of substrate, a main peak was detected for which the elution volume is compatible with the dimeric state of OBP. In addition, a minor shoulder was observed at higher elution volume suggesting the formation of a new oligomeric state of the pressure treated protein with an apparent molecular weight of 18.97 kDa, corresponding to the monomeric state. This shoulder was not detected in the presence of substrate, where exposure to 250 MPa did not induce any protein dissociation (data not shown). In contrast, upon exposure to 600 MPa, regardless of the absence or the presence of substrate, the recovered protein was found only in the monomeric state.

The chromatographic results are fully supporting the interpretation of the spectroscopic results. Interestingly, as summarized in Table 1, the pressure-induced spectral changes differed when recorded by increasing and decreasing pressure, indicating that the pressure-induced structural changes were partly irreversible. It appears that once the protein has lost the substrate at high pressure (above 350 MPa), a pressure release does not lead to substrate re-binding and to the protein dimerization. Only the refolding process at about 120 MPa can be observed. For elucidating the irreversible character of these processes, we carried out successive pressurization cycles of bOBP binding protein in the presence of substrate by recorded the protein fluorescence intensity emission at 338 nm. As shown in Figure 5A, the first pressurization cycle is characterized by a strong hysteresis behavior. This led to the very different p_1/2_ values between experiments with increasing and decreasing pressure, as noted in Table 1. In contrast, the profiles obtained from increasing and decreasing pressures in a second pressurization cycle did not show any hysteresis behavior. In fact, they were identical to the profile obtained with decreasing pressure in the first pressurization cycle. The initial p_1/2_ value of 320 MPa in the first cycle (at increasing pressure) was thus reduced to a p_1/2_ value of 150 MPa in the second cycle. From the size exclusion experiment we know that the dimer becomes irreversibly dissociated after exposure to 600 MPa. Furthermore, since the reduced p_1/2_ value is very similar to that of the engineered monomer in the absence of the substrate, the results are coherent with the proposed mechanism whereby the exposure of bOBP to 600 MPa led irreversibly to i) the lost of the bound substrate, and ii) to the dimer dissociation. The refolded protein (at atmospheric pressure) must be structurally different from the native protein, since it is no more capable of dimerization and substrate binding.

### Kinetics of the Pressure-induced Structural Changes

If the model described above is correct, this structural difference should also have a pronounced effect on the kinetics of the pressure-induced processes. Pressure jumps along the profiles of Figure 5A were hence used to investigate the kinetics of the pressure-induced structural changes of bOBP in the presence of the substrate. As shown in Figure 5B, the relaxation kinetics after upward p-jumps in the first pressurization cycle were complex. A very fast phase (τ <10 ms) was followed by a slow phase (τ ≈ 500 s) and finally by a very slow decrease of the fluorescence intensity. In contrast, in the second pressurization cycle the same p-jump induced very different kinetics: the first and very fast phase was absent, and the following phase was much faster (τ ≈ 100 s). The complexity of the dissociation/unfolding kinetics in the first pressurization cycle reflects probably the above mentioned hysteresis behavior. Conceivably, the loss of the bound substrate at high pressure is followed very rapidly, or is concerted with the dimer dissociation. The following slow phase would then represent the protein unfolding. For a reliable comparison of the two following pressurization cycles, we limited the analysis on the relaxation kinetics resulting from downward pressure-jumps. Figure 5C shows a comparison of the observed rate constants, *k*_obs_, measured along the profiles of the first and second pressurization cycles. The rate constants of the first and second pressurization cycles are rather similar. Since the equilibrium profiles were also very similar, this confirms that the protein, once it underwent a structural change by the first pressurization, it keeps this change, and it is no more affected by the second pressurization cycle.

Interestingly, there was a sharp dip of the speed of the reaction around 150 MPa that coincided with the p_1/2_ value of the equilibrium profiles. For further understanding the reasons of this dip, we carried out an analysis of the individual folding and unfolding rate constants, k_f_ and k_u_, as described in Material and Methods section. The results of this analysis are shown in Figure 5D. As expected from Figure 5C, it does not matter whether the rate constants were determined from the first or the second pressurization cycle. Furthermore, the representations of ln(k_f_) and ln(k_u_) as functions of pressure are straight lines, crossing at 180 MPa. The line of ln(k_f_) = f(p) has a strongly negative slope, that of ln(k_u_) = f(p) has a slightly positive slope. The origin of the sharp dip in Figure 5 is therefore explained by the fact that i) k_f_ is prevailing at low pressure and k_u_ at high pressure, and ii) ln(k_u_) = f(p) and ln(k_f_) = f(p) are straight lines of opposite signs, crossing approximately at the position of the dip. These straight lines can further be used to determine the corresponding activation volumes as ΔV^‡^_f_ = +95+/−8 ml/mol and ΔV^‡^_u_ = −5.5+/−0.7 ml/mol. The first value is rather large with respect to the latter. This indicates that the volume of the kinetic transition state is much closer to that of the folded than the unfolded state. This points to a relatively low kinetic stability of the folded monomer with respect to its unfolded form, because refolding would require large protein structural and/or hydrational changes. Apparently, dimerization and substrate binding appear to be the strategy of this lipocalin protein for overcoming this difficulty and increasing the structural stability.

### MD Simulation Experiments at High Pressure

Despite the low timescales with respect to experimental approaches, MD simulations were used since they are able to unravel molecular phenomena occurring to native bOBP in the presence of the ligand, when subjected to increasing pressures. In all simulations, the system was energetically stable, and the equilibration with respect to starting structure was reached at or before 10 ns, the simulation at 0.1 MPa showing the least RMSD value (data not shown). Moreover, periodic images were found to be at a distance of not less than 2 nm (higher than the cut-off used to evaluate electrostatic and van der Waals interactions) for the whole simulations. This assures that the simulation box is large enough to prevent periodic artifacts from occurring.

We analyzed the global features of bOBP structure submitted to the different pressures. The radius of gyration (R_g_) in all cases was found to be stable along the whole simulation, assuming a constant value of ∼2 nm. The analysis of clusters showed that few different clusters are present for each simulation. In particular, a single cluster of conformations is present in all simulations between 0 and 3 ns, indicating probably that, during this initial time, the systems reach their equilibration. For simulation at 0.1 MPa, 2 further main clusters of conformations are present: one (cluster 2) between 3 and 30 ns, and another one (cluster 1) between 10 and 100 ns. For simulation at 250 MPa, a single cluster of conformations (cluster 1) is present between 10 and 60 ns, coexisting with another cluster of conformations (cluster 2) between 60 and 86 ns. This last cluster becomes predominant between 86 and 100 ns. Remarkably, only one main cluster of conformations is present along the whole simulation at 600 MPa. The presence of multiple clusters of structures has been associated in the past to the presence of enhanced conformational variability and, possibly, to the existence of non-native conformations [46]. In this case, however, considering the extended length of simulation and the low number of different clusters, it is possible to conclude that the effect of pressure alone on protein’s tertiary structure is limited. The comparison of the representative structures of clusters (Figure 6) showed that the differences between them are focused mainly in the loops connecting the strands forming the central β-barrel. The analysis of root mean square fluctuations (RMSF) of residue’s positions (data not shown) pointed out that they are very similar in all simulations, and generally slightly more enhanced at 250 MPa with respect to other pressure values. Overall, these analyses confirm that pressure alone has a limited effect on the overall tertiary structure and dynamics of the protein.

The analysis of perturbations of secondary structures of the protein was made using DSSP along the whole simulation. Results are reported in Table 2 for the representative structures of the clusters. The content in β-sheet slightly decreases, and the percentage of residues in random coil slightly increases, at increasing pressure. However, the global effect of high pressure on the secondary structures of native bOBP seems to be quite limited.

Finally, further analyses were made in order to understand if pressure can perturb the dimeric assembly of the native protein. The analysis of the distances between the center of mass of the two subunits during the simulation time shows a limited decrease during the first part, followed by an increase of the distance, to recover the initial value (Figure 7). The average number of inter-chain contacts (atoms at a distance less than 2 nm) consistently increases from 1577.30 at 0.1 MPa to 1588.85 at 250 MPa to 1593.52 at 600 MPa, respectively. On the contrary, the number of inter-chain H-bonds in the dimer decreases from 248 to 239 to 169 at increasing pressures, and the average number of inter-chain H-bonds per frame decreases from 17.66 to 17.23 to 16.02 at 0.1, 250 and 600 MPa, respectively. The highest loss of H-bonds is present only at a pressure above 250 MPa, indicating that the protein in the presence of substrate is still relatively resistant to high pressure.

In conclusion, this analysis shows that in native conditions, bOBP has a strong intrinsic resistance to high pressure. MD simulations show that the interface between the two subunits is the part of the protein that undergoes the main perturbations. In sub-denaturing conditions, the first effect caused by pressure after loss of substrate is indeed the irreversible dissociation of the dimeric protein into monomers, followed then by reversible protein unfolding. Hence, dimerization and substrate binding significantly increase the resistance of the protein to pressure. These results overall confirm the fact that dimeric association and domain swapping can increase the resistance of the proteins towards external stresses.
